# Role demands and turnover intention among Covid-19 frontline nurses: The mediating and moderating roles of compassion fatigue and spiritual leadership

**DOI:** 10.1371/journal.pone.0289888

**Published:** 2023-08-10

**Authors:** Arielle Doris Tetgoum Kachie, Lulin Zhou, Prince Ewudzie Quansah, Xinglong Xu, Thomas Martial Epalle, Berty Nsolly Ngajie

**Affiliations:** 1 Centre for Medical Insurance, Hospital Management and Health Policy Research, School of Management, Jiangsu University, Zhenjiang, Jiangsu province, China; 2 School of Management, Jiangsu University, Zhenjiang, Jiangsu province, China; 3 Department of Computer Engineering, School of International Business, Zhejiang International Studies University, Hangzhou, Zhejiang province, China; 4 School of English Language and Culture, Zhejiang International Studies University, Hangzhou, Zhejiang Province, China; Universidad Central de Chile, CHILE

## Abstract

The working conditions created by the Covid-19 pandemic have been proven to amplify frontline nurses’ desire to leave their profession in recent years; thus, exploring new causing variables is vital. This cross-sectional study examined role demands’ direct and indirect effects on turnover intention through compassion fatigue and tested the various dimensions of spiritual leadership as moderators on the relationship between compassion fatigue and turnover intention. A total of 527 valid responses were collected from frontline nurses working in designated hospitals across Zhejiang province in China using a survey questionnaire. The outcomes from the hierarchical regression analysis indicated that role demands positively and significantly impacted turnover intention and compassion fatigue. Besides, compassion fatigue significantly and positively affected turnover intention and mediated its relationship with role demands. However, vision and altruistic love moderated the relationship between compassion fatigue and turnover intention, which was not true for the dimension hope/faith. This study’s findings are a steppingstone for medical firms’ managers and policymakers in demonstrating the likelihood of frontline nurses developing turnover thoughts arising from ambiguous and conflicting roles and the emotional strain from patients’ burdens. Furthermore, an exemplary implementation of spiritual leadership could help enhance nurses’ sense of calling and membership, essential in embracing the organization’s vision and achieving its goals.

## Introduction

The world has experienced several pandemics, but the ongoing Covid-19 pandemic has threatened global health for over three years. Medical systems continue to be tested, affecting the medical profession agents, especially frontliners. The current context has already been proven to affect the leave of medical agents, and the factors involved are becoming more diverse [1]. Besides, interest in nursing turnover has never been that high compared to the last three years [2, 3]. Since the actual turnover is not easy to ascertain among current employees, the turnover intention has usually been considered a proxy to estimate workers’ propensity to turn over their present job position [4].

The relevance of turnover intention in increasing the nursing shortage makes it vital to develop programs and systems to enhance nurse retention. In doing so, medical organizations may improve competitiveness, productivity, and service delivery in challenging times [5, 6]. Past research has exposed the variety of factors affecting turnover intention. Some of these factors are stress factors [7], employees’ attitudes such as satisfaction, commitment, engagement, and safety performance [1, 8, 9], and employees’ characteristics such as morale, resilience, agility, and organizational aspects [10, 11]. Nevertheless, under the current condition of Covid-19, new challenges have arisen, the effects of most of these factors may not be the same, and new elements might have come into action. For example, role demands are regarded as the uncertainties workers face when performing tasks. The directions can be contradictory when they come from different superiors (role conflict) or confusing when they lack clarity (role ambiguity), which may induce a certain amount of stress on the employees [12]. It is clear that with the changing nature of the pandemic, primarily due to the constant new variants, the demands on nurses may sometimes be ambiguous or unclear. It is, therefore, essential to explore how role demands may influence turnover intention among Covid-19 frontline nurses.

Moreover, the caring nature of nursing encapsulates compassion that can result in satisfaction or exposure to physical and emotional distress (compassion fatigue) [13]. The calling upon nurses satisfies them, especially when they can provide the necessary care to their patients [5, 14]. However, compassion fatigue occurs when the connection built with the suffering person during the caring process crushes their level of empathy to the point that they are drained emotionally and physically and tend to lose their ability to nurture [15]. It has also been proven that being unable to complete the different assignments or finding ambiguities in the orders received from several superiors seriously affects the caregiver’s providing care attitude and abilities [13, 16]. Understanding the new influential causing factors of the nursing turnover intention in investigating how role demands could explain the turnover intention of frontline nurses is vital. Thus, this study also finds it relevant to examine how compassion fatigue might mediate that relationship.

In challenging times, if the company is aware that workers find their work demands troublesome, are dealing with a certain level of trauma or burnout, and are constantly thinking of leaving, the appropriate response is to find mitigative solutions. A good leadership style has been shown to bring the best out of each composite of the work team [17, 18]. Spiritual leadership is of growing interest in medical institutions because it emphasizes improving employees’ quality care delivery to a more professional level while nurturing their well-being and increasing patients’ satisfaction [19]. Caring for suffering people is difficult, so nurses need to develop a healthier inner life that will help them stay balanced and have meaningful work [17]. Spiritual leadership not only builds workers’ intrinsic motivation but also creates a favorable ground for them to identify with the vision and goals of the company efficiently. In such a working environment, employees hardly allow thoughts of leaving but are willing to give their best to benefit the organization’s growth and clients’ satisfaction [20]. Unfortunately, the integration of spiritual leadership is still emerging in the medical world, making the related literature sparse in that research area [19]. This study thus proposes to apply spiritual leadership as a moderator on the mediating mechanism (compassion fatigue and turnover intention) to see the changes it may produce.

The Job Demands-Resources (JD-R) theory [21, 22] was found more suitable to explain the hypothesized relationships in this study. The theory posits that job characteristics affect workers’ well-being and efficiency in any given job. Therefore, it is assumed that job demands and job resources characterize any position. Job demands include those physical, social, or organizational aspects of the job that require continuous physical, emotional, and mental endeavors, such as high workload, job insecurity, and conflicting and ambiguous demands from supervisors or recipients. However, job resources are those aspects of the job destined to enhance work goals achievement, decrease job demands and associated costs, and stimulate personal growth and development. Examples of job resources include autonomy, social support, promotion, and feedback.

**Fig 1:**
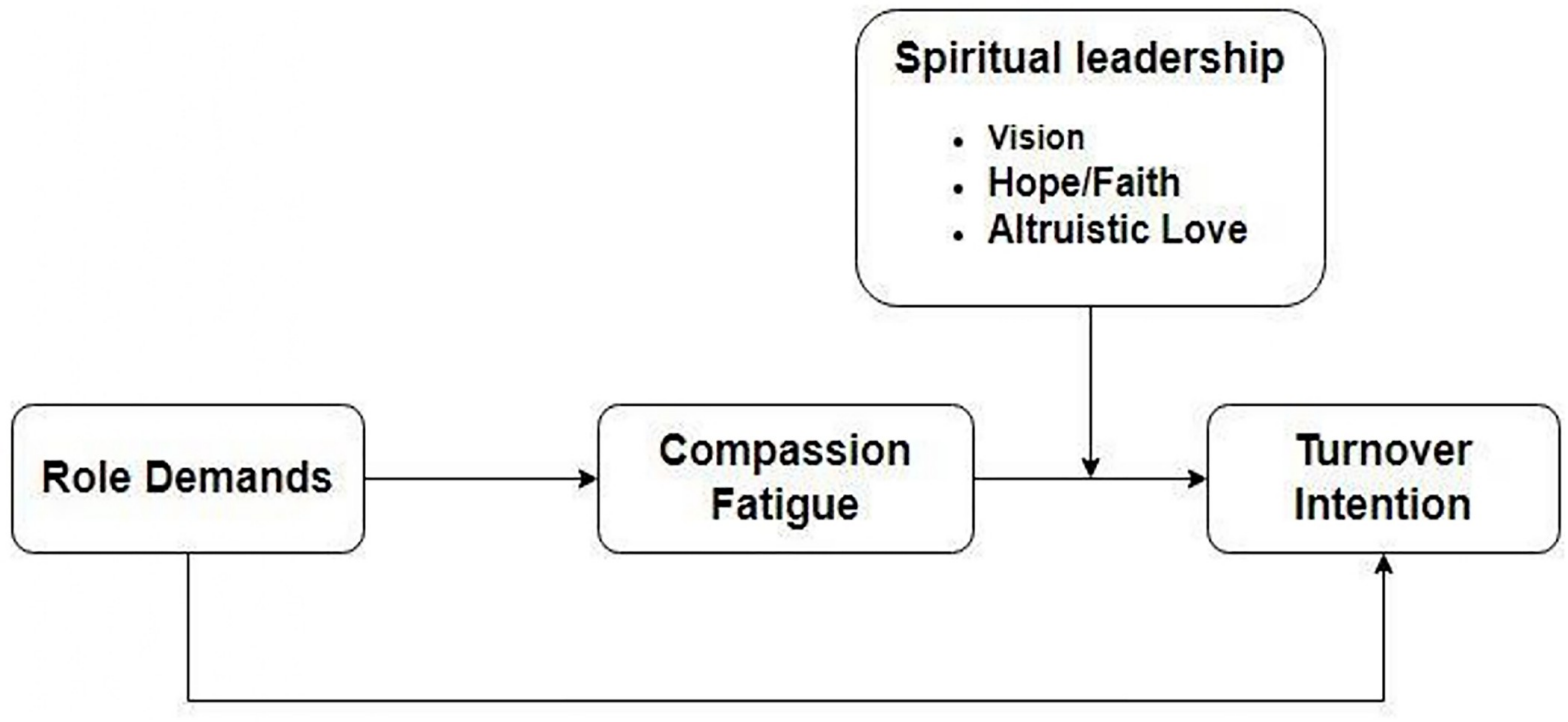
Conceptual framework.

The JD-R stipulates that when job demands are not proportional to job resources, on the one hand, workers are overtaxed, tired, and irritated, which can easily lead to exhaustion. On the other hand, the lack of resources does not help meet the high demands, which affects their health-protecting mechanism and does not favor achieving work goals. In such a situation, individuals are demotivated and generally choose withdrawal behavior to avoid future frustrations [21, 22]. The theory also proposes that additional effort must be applied to mitigate the negative influence of job demands on employees’ performance and productivity [23]. The assumptions on which the theory rests, thus, give more ground to its application in this study. This research, therefore, tempts to investigate how far role demands could influence the turnover intention of Covid-19 frontline nurses through compassion fatigue. It also explores the intervention of spiritual leadership as a moderator on the relationship between compassion fatigue and turnover intention. The conceptual framework showing the linkages among the different variables involved is displayed in Fig 1.

## Literature review and hypotheses development

### Turnover intention

Turnover intention is gradually regarded as a bottleneck for medical institutions’ growth because of its increasing propensity [2]. It refers to individuals’ strong desire to forfeit their job position in an organization [24]. According to Reference [25], total resignation is a gradual process that starts with the thought of leaving and is followed by the intention to look for a new job. Finally, the employee will actively invest in the intention to leave the job.

With the demanding nature of their career, nurses are more prone to think of leaving. Evidence suggests that the Covid-19 context has increased the propensity of frontline nurses to turn over their job under the influence of various factors [1]. Though employees’ well-being is trivial for staying focused and continuously willing to achieve their organizational objectives, it may be influenced by the job’s requirements and resources [23]. When workers notice a decrease in their satisfaction or a significant loss of energy in a high job demand and poor job resources context, they tend to huddle up on themselves to protect their intrinsic resources for future and better investment. The situation can be avoided by putting in more effort to turn back and stabilize the situation [26]. Generally, in such a situation, turnover intention may occur [27]. The working conditions of frontline nurses during the Covid-19 pandemic offer an interesting ground to understand the new variables that could instigate them to think of leaving.

### Role demands and turnover intention

The work-task-related difficulties and the uncertainties associated with a particular job position under certain conditions have been regarded as role demands. In the organizational design and job stress literature, role demands are considered a source of stress and consist of role conflicts and ambiguity [8]. Role conflicts happen when the chain of command does not respect the flow of authority and the sources of orders are diverse; the employee becomes caught in the crossfire of incompatible demands or expectations from more than one superior. However, role ambiguity occurs when the individual finds the order unclear, making it difficult to perform [28]. In addition, an organization’s hierarchical relationship is set up so that the group of activities should point to one common objective and be directed by only one leader.

Moreover, for coordination and control to be more effective, workers must be accountable for successfully implementing their tasks to only one leader [12]. One thing with the Covid-19 pandemic is its changing nature, which implies continual changes in the protocols and assessment of the workforce’s adaptability to new terms and demands. These conditions might inevitably violate communication and create an unstable working milieu. The incompatibility and the lack of clarity in the sent roles have been proven to induce stress, which might increase the likelihood of workers being dissatisfied with their roles, anxious, performing less effectively, and eventually having thoughts of leaving [8]. This idea is emphasized in the JD-R theory when it argues that there is a relationship between job demands, resources, and employees’ well-being and organizational outcomes. The balance between job requirements and resources foster positive job attitudes (e.g., satisfaction, engagement, commitment) and goal achievement and reduces withdrawal behaviors (e.g., absenteeism, turnover intention, and actual turnover) [27]. We, therefore, lean on the JD-R theory and other related literature to propose the following hypothesis:

H1: Role demands will significantly and positively influence turnover intention.

### Role demands and compassion fatigue

As in many studies, this study will identify compassion fatigue as a combination of secondary trauma and burnout [13, 15, 29]. Compassion fatigue happens more in caregiving professions and occurs when individuals are no more enthusiastic nor fulfilled when helping others but are negatively affected by what their clients went or are going through instead [15]. Providing care to traumatized or suffering persons may overlap the caregivers’ capacities such that they start showing reduced empathy until emotional exhaustion [30]. Nursing has always been a straining profession because nurses can easily experience physical and psychological fatigue [13]. Moreover, prolonged and intense contact with suffering patients may devastate them, primarily when they cannot provide appropriate care, which might be detrimental to the patients and the organization [31]. As Alharbi, Jackson, & Usher (2020) described, the emotional strain and the patients’ burden may create a dysfunctional and exhaustion state because it is easy to absorb the helping person’s traumatic stress when caring and showing empathy.

During this Covid-19 pandemic, hospitals have been pressed with sick people, increasing the workload and threatening available resources [32]. Caring for distressed people may be highly stressful for nurses in such a working environment. Nurses have to use themselves and their skills to fulfill the need of their numerous patients [33, 34]. The dying ones sometimes require intense emotional support [35]. Evidence suggests that exposure to such a working environment for a long time might affect the nurses’ caring capacity, especially when they fail to provide such aid [34]. In addition, the uncertainty of the disease favors the fact that instructions possibly coming from different authorities could be confusing and unclear. Then, nurses might be unable to cope with the caregiving demands, which may increase their likelihood of developing compassion fatigue [36]. Such conditions lead to decreased attention span, reduced delicacy, discomfort, and a sense of weariness, and the pleasure of serving in their profession is fading away [37]. The unpredictable characteristics of the working environment have been shown to amplify role conflict and ambiguity [12, 38]. Besides, Reference [36] demonstrated a positive influence of role conflict and ambiguity on compassion fatigue.

Moreover, drawing on JD-R theory, it is stipulated that long-term high job demands require additional effort to achieve organizational goals and good performance. Eventually, fatigue and irritability are examples of physical and psychological costs that will follow, which may gradually exhaust workers [21]. From this perspective, we, therefore, hypothesize the following:

H2: Role demands will significantly and positively impact nurses’ compassion fatigue.

### Compassion fatigue and turnover intention

Caring for suffering people can be costly because compassion and the desire to relieve others’ sufferings may bring pain to the caregiver with limited abilities [39]. The prevalence of compassion fatigue is proven to be high in nursing, having detrimental impacts on nurses’ physical, mental, and spiritual well-being as well as on their practice [40]. The new challenges experienced by nurses during the Covid-19 pandemic have provoked psychological distress, causing them to be more vulnerable and susceptible to compassion fatigue [39]. Compassion fatigue creates disorder in oneself, causing careless mistakes, which is dangerous for nurses and their patients. Furthermore, the gap between the stress level and the endurance limits overwhelms them emotionally to the extent that rest is no longer a solution [41]. In their study, Reference [42] argued that compassion fatigue might considerably cause increased burnout for nurses, low productivity, and influence their desire to stay in their job. The JD-R theory corroborates this reasoning when it postulates that excessive and long-term job demands from which workers do not recover properly are overtaxing for them and usually lead to exhaustion [22, 23]. Parallelly, when the available resources are insufficient to mitigate the negative influence of job demands on exhaustion, employees start showing decreased motivation and engagement, leading to withdrawal behaviors [43]. Indeed, when facing compassion fatigue, employees just want to quit as a self-protective measure to prevent more energy depletion and avoid such a situation again [42]. Based on evidence from previous research and dragging on the JD-R theory, this study hypothesizes:

H3: Compassion fatigue affects nurses’ turnover intention significantly and positively.

### Compassion fatigue as a mediator

A mediator variable explains and clarifies the nature of the relationship between an independent variable and an outcome variable [44]. The study assumes that the effect of role demands on turnover intention is better explained when in between, compassion fatigue is applied. The above literature review discloses the influence of role demands on compassion fatigue and turnover intention. The impact of compassion fatigue on turnover intention in organizations was also made clear. Although these three factors are interrelated, their linkage is scarce in the extant literature. Compassion fatigue encapsulates secondary traumatic stress and cumulative burnout. Moderate to high rates of compassion fatigue has been highlighted in recent studies among nurses, resulting in an adverse impact on the individual’s mental health and well-being and negative organizational outcomes [45–47]. Moreover, JD-R theorists argue that a work environment with challenging job demands easily foster exhaustion, which is the dynamic aspect of burnout. Also, if adequate resources are not provided to reverse the situation and allow workers to achieve their organizational goals, they are tempted to retract themselves from the company’s activities [23]. Almost four years of the Covid pandemic is an example of excessive and continual exposure to danger for frontline nurses, which is traumatizing and threatens nurses’ well-being [46, 47]. From this premise, we hypothesize:

H4: Compassion fatigue will significantly mediate the relationship between role demands and turnover intention.

### Spiritual leadership as a moderator

Spirituality is a growing concern in nursing literature [17, 19]. It helps employees question life’s meaning and nurture their desire to connect more with their organization’s goals and have meaningful work [48]. With the current Covid-19 pandemic context, nurses’ daily challenging working conditions are not to be demonstrated anymore. Researchers opinionated that individual factors, such as spirituality, and contextual factors, such as leadership, can help curve the negative effects on nurses’ quality of care and well-being [49]. A good leadership style is thus critical in assisting nurses in satisfying their psychological needs, having a strong conscience of their professional calling, and being fulfilled at work [50]. Therefore, implementing spiritual leadership is increasingly promoted in healthcare institutions [48, 49].

Nonetheless, few empirical studies have shown how it can alter the relationship between two other variables as a third variable. Spiritual leadership intrinsically motivates both leaders and followers through the values, attitudes, and behaviors it promotes. In that way, it ensures the spiritual survival needed for calling (the ability to serve others selflessly) and membership (having a sense of belonging and a community-based orientation) [51]. In addition, it incorporates vision, hope/faith, and altruistic love. Inherently, the sense of calling helps leaders and followers to embrace the organization’s vision and work towards excellence to make a difference [52, 53].

Moreover, an organizational culture built on altruistic love values is the proper ground to develop leaders’ and followers’ sense of membership, where mutual understanding, appreciation, care, and concern are shared [17]. Therefore, workers see meaning in their job and are willing to work hard to be more productive and achieve their best performance. Additionally, they are not afraid to express themselves sincerely, believing in their company to ensure their interests [19]. Indeed, as Yang & Fry (2018) and Saleh et al. (2018) highlighted, appropriate leadership can help rebuild a positive work environment that could alleviate the negative effect of burnout and traumatic stress and consequently contribute to workers’ retention. The previous argumentation meets the predictions of the JD-R theory when it stipulates that additional resources play external and internal motivational roles. These resources may buffer the adverse impact of high job demands on workers’ negative psychological state [16, 43]. Adequate job resources decrease job demands and associated costs by enhancing workers’ fulfillment and positive state of mind. Besides, when job demands are reduced, individuals can easily see their fundamental human needs for competence, autonomy, and relatedness being satisfied, which fosters their willingness to put additional effort into achieving organizational goals [23]. Towards this light, we propose the following hypotheses:

H5: Spiritual leadership will significantly moderate the relationship between compassion fatigue and turnover intention.

H5a: Vision will significantly moderate the relationship between compassion fatigue and turnover intention.

H5b: Hope/Faith will significantly moderate the relationship between compassion fatigue and turnover intention.

H5c: Altruistic love will significantly moderate the relationship between compassion fatigue and turnover intention.

## Materials and methods

### Design and settings

This cross-sectional study gathered data from frontline nurses working in the designated hospitals for diagnosing and treating pneumonia caused by the novel coronavirus across Zhejiang province in China. Zhejiang province is among the leading regions in China, covering 105,500 km^2^ with a population of about 65.4 million spread over 11 prefecture-level cities [54]. Given its large population, 100 hospitals were designated across the 11 prefectures to manage patients with Covid-19, having four at the provincial level. They are primarily third-level (with more than 500 beds) and second-level (with 100–500 beds) hospitals. Though these hospitals do not have the same capacity, the government took measures to equip them similarly and adequately to handle Covid-19 cases successfully [55].

Invitations were randomly sent to participants at 50 hospitals in all 11 cities. As a result, the 23 hospitals that agreed to participate were distributed as follows: one at the provincial level, two in Hangzhou, one in Huzhou, two in Shaoxing, two in Jinhua, three in Taizhou, three in Ningbo, two in Wenzhou, two in Jiaxing, one in Zhoushan, two in Quzhou, and two in Lishui.

### Data collection

After receiving approval from the various hospitals’ authorities, one nurse was recommended in each hospital to accompany us in the data collection process. China can be identified as a high-tech country, and in their daily life, people use their phones to access information and handle many activities. An online approach was then chosen to collect data because it is more convenient and fits better with the pandemic’s context and restrictions. Short meetings were held online with the nurses’ representatives to explain the issues of the study and the nature of the questionnaire consisting of four standardized scales and demographic variables such as gender, age, education level, and professional experience. They were informed about the study’s objectives and encouraged to participate, as the results could help improve the health system and their working conditions. Although they were encouraged this way, we told them that participation is voluntary and anonymous, that their answers cannot be disclosed but only used in this research and that they are free to decline their participation at any time. It was also important to inform them that the questions were designed to be all answered and that they only had to choose the best answer that suited their situation. During the study period, other meetings with nurses’ representatives were organized halfway through and just before the closure to make adjustments and improve participation. As suggested in previous studies, this process was essential to manage invalid responses and biases [56–58].

As a whole, 1000 registered frontline nurses in service during the survey with a working experience of six months or above were invited to answer the survey questionnaire between January to March 2022, generally a period of increase in Covid-19 cases. Only registered Covid-19 frontline nurses were eligible to participate. Those absent or on leave during the survey and those who have not been directly involved for at least six months in caring for patients with coronavirus in their actual work unit were not. Out of 1000, 527 responses were received, representing 52.7% of the response rate. Of the 527 respondents, 341 (72.3%) were females, and 146 (27.7%) were males. In addition, 208 (39.5%) of the participants were between 20–30 of age, 201 (38.5%) between 31–40 and 116 (22%) between 41–50. Moreover, regarding their educational level, 319 (60.5%) had a university education, and 208 (39.5%) had vocational education. Also, only 11 (2.1%) had been working for less than a year, 116 (22%) had worked for 1 to 5 years, 150 (28.5%) had worked for 6 to 10 years, and 250 (47.4%) had worked for more than ten years.

### Ethics statements

The Ethical Review Board of Jiangsu University approved this study involving human participants, with the approval number JU-EBR-15/12/21. The participants also provided their written informed consent to participate in this study.

### Instrumentation

Regarding the respondents’ primary language, the whole questionnaire (see S1 Appendix) was translated into “Mandarin,” the official Chinese language regarding the state of the art, as suggested by Reference [59]. After the tool’s content validity was certified by a few members of the postdoctoral team, the questionnaire was tested on 30 nurses recruited only for that purpose since they were not part of the final study. Their remarks helped nullify any misunderstanding and polish the questions.

Moreover, based on recommendations from previous studies [8, 60], a single mean score was calculated for role demands and compassion fatigue using the averages of their subscales’ items. This process was adopted because their dimensions were highly correlated with each other.

#### Role demands

Role demands were assimilated as role conflict and role ambiguity in this study. Role demands were gauged by the 14 items of the role questionnaire developed by Reference [12]. Of the 14 items, height was used to measure role conflict (e.g., I receive incompatible requests from two or more people) and six for role ambiguity (e.g., Explanation is clear of what has to be done). Previous research established the scale’s internal consistency, ranging from 0.874 to 0.966 [8]. The total Cronbach’s Alpha value was 0.882 in this study, with 0.924 and 0.905 for role conflict and role ambiguity, respectively. The items were measured on a seven-point Likert scale ranging from 1 (strongly disagree) to 7 (strongly agree), with higher scores indicating a higher demanding role in conflict and ambiguity.

#### Compassion fatigue

Nurses’ compassion fatigue was assessed using the 13-item short version of Reference [30]. The scale was judged valid and reliable (α = 0.90 [30]) and has been widely used to identify social workers exposed to secondary trauma (five items (α = 0.901), e.g., Intrusive thoughts after working with difficult clients) and job burnout (eight items (α = 0.906), e.g., Felt tired due to work as a caregiver). Nurses answered compassion fatigue items using a seven-point Likert scale from 1 (never) to 7 (very frequent). In this study, the items’ reliability result was 0.873, and an increase in the score reflected a higher degree of compassion fatigue.

#### Spiritual leadership

Spiritual leadership was operationalized using the 17-item tool Fry, Vitucci, & Cedillo (2005) proposed. It incorporates vision (5 items, α = 0.967), hope/faith (5 items, α = 0.892), and altruistic love (7 items, α = 0.941). Examples of items stated: “My work group has a vision statement that brings out the best in me” (vision); “I persevere and exert extra effort to help my organization succeed because I have faith in what it stands for” (hope/faith); “My organization is kind and considerate toward its workers, and when they are suffering, wants to do something about it” (altruistic love). The scale has demonstrated good internal consistency and validity in this study, with overall Cronbach’s alpha being 0.948. In addition, all the items were estimated on a seven-point Likert scale ranging from 1 (strongly disagree) to 7 (strongly agree), with a high score indicating a greater influence of spiritual leadership implementation.

#### Turnover intention

Turnover intent was measured by three items developed by [61]. The items used a seven-point Likert scale, ranging from 1 (strongly disagree) to 7 (strongly agree), with a higher score representing an increased desire to leave the organization (e.g., I often think about leaving the organization). The turnover intention was used in this study instead of actual turnover as it is easier to measure, partly because it is assessable among current rather than former employees [4]. The original internal consistency was good, 0.83 [61], and in the current study, it was 0.921.

### Data analysis

This study used two data analysis tools, SPSS version 26 and AMOS version 22. SPSS was used to perform the data’s defining aspects and the exploratory factor analysis (EFA). It was also used later to perform hierarchical regression to test the various path analysis. Meanwhile, AMOS was used to carry out confirmatory factor analysis (CFA) and provide extra support to the data set.

## Results

### Unidimensionality, reliability, and validity of the variables

The variables’ unidimensionality was established in this study by checking the combination of their comparative fit index (CFI), root mean square error of approximation (RMSEA), and standardized root mean square residual (SRMR), as proposed by Reference [62]. All four variables demonstrated a CFI (0.949) > 0.90, RMSEA (0.044) < 0.06, and SRMR (0.033) < 0.08 as the suggested cutoff criteria. The appropriateness of the data was established as well, and the values of Kaiser–Meyer–Olkin measure of sampling adequacy (KMO-MSA) (0.928) and Bartlett’s test of sphericity (BTS) (X2 = 20462.325; df = 1081; p < 0.001) were within acceptable levels.

The exploratory factor analysis (EFA) was performed in SPSS. The results showed that all items were loaded under their relevant factor and had loadings greater than the 0.50 rule of thumb [63, 64]. After that, the scales’ internal consistency was checked through Cronbach’s Alpha. As seen in Table 1, all the various constructs had an α-value greater than the 0.70 suggested value. Moreover, the variables’ factor loadings from the confirmatory factor analysis (CFA) were above 0.5 thresholds and significant at a p-value < 0.001 (see Table 1).

**Table 1:** CFA factor loadings, reliability, and validity results.

Constructs	Dimensions	Items	Factor loadings	S.E.	C.R.	α	CR	AVE
Role Demands	Role Conflict (RC)	RC8	0.859			0.924	0.924	0.604
		RC2	0.791	0.041	22.23			
		RC7	0.775	0.042	21.518			
		RC5	0.75	0.042	20.465			
		RC1	0.785	0.042	21.978			
		RC3	0.755	0.041	20.669			
		RC4	0.764	0.042	21.078			
		RC6	0.731	0.043	19.71			
	Role Ambiguity (RA)	RA4	0.964			0.905	0.907	0.621
		RA1	0.768	0.034	24.582			
		RA6	0.722	0.036	21.827			
		RA5	0.758	0.035	23.978			
		RA3	0.793	0.034	26.305			
		RA2	0.697	0.037	20.533			
Compassion Fatigue	Job Burnout (JB)	JB5	0.906			0.901	0.911	0.566
		JB8	0.87	0.033	28.958			
		JB3	0.744	0.04	21.498			
		JB4	0.738	0.037	21.185			
		JB7	0.711	0.042	19.938			
		JB2	0.709	0.043	19.855			
		JB6	0.658	0.042	17.709			
		JB1	0.643	0.055	17.114			
	Secondary Trauma (ST)	ST3	0.841			0.906	0.901	0.646
		ST2	0.841	0.041	23.019			
		ST5	0.82	0.043	22.2			
		ST4	0.754	0.045	19.673			
		ST1	0.758	0.044	19.81			
Spiritual Leadership	Vision (VI)	VI3	0.936			0.967	0.967	0.853
		VI4	0.933	0.025	41.391			
		VI2	0.925	0.025	40.046			
		VI5	0.925	0.025	40.141			
		VI1	0.898	0.027	36.239			
	Hope/Faith (HF)	HF3	0.902			0.892	0.929	0.728
		HF2	0.898	0.031	32.108			
		HF5	0.898	0.031	32.127			
		HF4	0.914	0.03	33.642			
		HF1	0.616	0.073	16.343			
	Altruistic Love (AL)	AL5	0.893			0.941	0.941	0.697
		AL4	0.896	0.031	31.02			
		AL2	0.856	0.033	28.051			
		AL7	0.818	0.034	25.541			
		AL6	0.816	0.036	25.433			
		AL1	0.783	0.034	23.549			
		AL3	0.772	0.035	22.949			
Turnover Intention	Turnover Intention	TI1	0.913			0.921	0.921	0.796
		TI2	0.91	0.032	31.02			
		TI3	0.852	0.034	27.614			

**Abbreviations**: S.E., Standard Error

C.R., Critical Ratio

α, Cronbach’s Alpha

CR, Composite Reliability

AVE, Average Variance Extracted.

More attention was given to the constructs’ validity. The AMOS plugin developed by Reference [62] helped generate the values of the composite reliability (CR), average variance extracted (AVE), discriminant validity (DV), and correlation table automatically using standardized coefficients and correlation values. The various constructs’ convergent validity was good because all the CR and AVE values exceeded the 0.70 and 0.50 cutoffs, respectively, as proposed by Reference [65] (see Table 1).

Table 2 presents the DV results. The bold values along the diagonal line of the inter-factor correlation matrix show that though the variables are related, they are distinct because their correlation coefficients are higher than those of their corresponding inter-factor. Furthermore, a CFA model fit comparison test was done to establish the best model fit for the data set. As displayed in Table 2, all the models had good fit statistics. However, the 6-factor model best fits the data set compared to a 1-factor, 4-factor, 5-factor, 7-factor, and 8-factor model. The 6-factor reported a Chi-square statistics (X2 = 2037.932), normed Chi-square fit index (X2/ df) = 2.026, comparative fit index (CFI) = 0.949, Tucker–Lewis fit index (TLI) = 0.945, and goodness of fit index (GFI) = 0.876. Likewise, the values of the standardized root mean square residual (SRMR) = 0.033 and root mean square error of approximation (RMSEA) = 0.044 were within the acceptable levels, <0.08 and <0.06.

**Table 2:** Model fit comparison.

Models	X^2^	X^2^ /df	SRMR	RMSEA	CFI	TLI	GFI
8-Factor model	2062.979	2.032	0.041	0.044	0.940	0.934	0.874
7-Factor model	2057.407	2.035	0.039	0.044	0.948	0.944	0.875
7-Factor model	2054.681	2.032	0.04	0.044	0.948	0.944	0.875
6-Factor model	2044.904	2.013	0.034	0.044	0.949	0.945	0.875
6-Factor model	2037.932	2.026	0.033	0.044	0.949	0.945	0.876
5-Factor model	2060.336	2.022	0.04	0.044	0.938	0.931	0.874
5-Factor model	2060.257	2.022	0.048	0.053	0.926	0.917	0.874
4-Factor model	2065.081	2.023	0.051	0.059	0.908	0.895	0.874
1-Factor model	2148.592	2.094	0.058	0.076	0.901	0.884	0.869

**Notes**: 8-Factor model (Role conflict, Role ambiguity, Secondary trauma, Job burnout, Vision, Hope/Faith, Altruistic love, Turnover intention); 7-Factor model ((Role conflict and Role ambiguity combined), Secondary trauma, Job burnout, Vision, Hope/Faith, Altruistic love, Turnover intention); 7-Factor model (Role conflict, Role ambiguity, (Secondary trauma and Job burnout combined), Vision, Hope/Faith, Altruistic love, Turnover intention); 6-Factor model (Role conflict, Role ambiguity, Secondary trauma, Job burnout, (Vision, Hope/Faith and Altruistic love combined), Turnover intention); 6-Factor model ((Role conflict and Role ambiguity combined), (Secondary trauma and Job burnout combined), Vision, Hope/Faith, Altruistic love, Turnover intention); 5-Factor model ((Role conflict and Role ambiguity combined), Secondary trauma, Job burnout, (Vision, Hope/Faith, and Altruistic love combined), Turnover intention); 5-Factor model (Role conflict, Role ambiguity, (Secondary trauma and Job burnout combined), (Vision, Hope/Faith, and Altruistic love combined), Turnover intention); 4-Factor model ((Role conflict and Role ambiguity combined), (Secondary trauma and Job burnout), (Vision, Hope/Faith and Altruistic love), Turnover intention); 1-Factor model (All variables combined); X^2^, Chi-square; X^2^ /df, normed Chi-square fit index; SRMR, standardized root mean square residual; RMSEA, root mean square error of approximation; CFI, comparative fit index; TLI, Tucker–Lewis incremental fit index; GFI, the goodness of fit index.

### Means, standard deviation, and correlation analysis

Table 3 presents the measures’ means, standard deviation, and inter-factor correlation analysis. The correlation analysis results show that all two components of role demand significantly and positively correlated with turnover intention and compassion fatigue, suggesting some initial support for H1 and H2. Also, compassion fatigue dimensions positively correlate with turnover intention, offering preliminary support for H3.

**Table 3:** Inter-factor correlation analysis.

	Mean	SD	AL	RC	JB	VI	RA	ST	HF	TI
**Gender**	1.28	0.448								
**Age**	1.83	0.765								
**Education**	1.61	0.489								
**Experience**	3.21	0.857								
**AL**	3.4584	1.55439	**0.835**							
**RC**	4.1928	1.46525	-0.105*	**0.777**						
**JB**	4.4371	1.2724	-0.245***	0.232***	**0.752**					
**VI**	3.4565	1.75752	0.499***	-0.173***	-0.272***	**0.923**				
**RA**	4.0588	1.33528	-0.234***	0.192***	0.307***	-0.236***	**0.788**			
**ST**	3.7359	1.28616	-0.150**	0.065	0.219***	-0.181***	0.393***	**0.804**		
**HF**	2.9662	1.61028	0.649***	-0.244***	-0.335***	0.687***	-0.328***	-0.254***	**0.853**	
**TI**	3.9165	1.61118	-0.284***	0.103*	0.232***	-0.316***	0.273***	0.263***	-0.373***	**0.892**

**Notes**: *p < 0.05, **p < 0.010, *** p < 0.001; The bolded values represent discriminant validity

Abbreviations

AL, Altruistic love

RC, Role conflict

JB, Job burnout

VI, Vision

RA, Role ambiguity

ST, Secondary trauma

HF, Hope/Faith

TI, Turnover intention.

### Hypotheses testing

#### Testing the main effect and mediating effect of compassion fatigue

The main and mediating effects hypotheses were estimated through hierarchical regression in SPSS while controlling for gender, age, education, and experience. The results are displayed in Table 4. From Model 2, it can be seen that role demands (β = 0.324, p < 0.001) significantly and positively predicted nurses’ turnover intention; hence H1 was supported. Model 3 similarly shows that role demands (β = 0.345, p < 0.001) positively and significantly influenced compassion fatigue, favoring H2. Model 4 in Table 4 also demonstrates that compassion fatigue (β = 0.487, p < 0.001) had a significant impact on turnover intention, making H3 supported. Concerning the mediating effect of compassion fatigue between role demands and nurses’ turnover intention relationship, Model 5 in Table 4 reveals that when compassion fatigue is applied between the two variables, nurses’ turnover intention is still significantly predicted by role demands (β = 0.182, p < 0.01). Moreover, compassion fatigue (β = 0.413, p < 0.001) could again affect turnover intention significantly, implying a partial mediation for compassion fatigue in that relationship. H4 was then favorable too.

**Table 4:** Hierarchical regression analysis results of the main effect and the mediating effect of job engagement.

Variables	Turnover intention	Turnover intention	Compassion fatigue	Turnover intention	Turnover intention
	Model 1 *β* (t)	Model 2 *β* (t)	Model 3 *β* (t)	Model 4 *β* (t)	Model 5 *β* (t)
First step (Control variables)					
Constant	4.266*** (6.196)	3.036*** (4.241)	2.631*** (6.346)	2.346*** (3.298)	1.95** (2.7)
Gender	0.064 (0.294)	0.039 (0.186)	0.007 (0.056)	0.048 (0.23)	0.037 (0.177)
Age	-0.065 (-0.699)	-0.074 (-0.818)	-0.074 (-1.42)	-0.033 (-0.376)	-0.043 (-0.492)
Education	-0.05 (-0.344)	-0.07 (-0.487)	-0.101 (-1.223)	-0.011 (-0.078)	-0.028 (-0.2)
Experience	-0.072 (-0.63)	-0.081 (-0.724)	0.1 (1.54)	-0.126 (-1.143)	-0.122 (-1.121)
Second step (Main effect) Role Demand		0.324*** (5.036)	0.345*** (9.26)		0.182** (2.691)
Third step (Mediation analysis) Compassion fatigue				0.487*** (7.108)	0.413*** (5.614)
R^2^	0.003	0.05	0.153	0.091	0.104
Δ R^2^	0.003	0.046	0.139	0.088	0.101
F	0.435	11.438***	28.799***	21.486***	17.05***

**Notes**: *β*, Unstandardized estimates; (t), t-values; *p < 0.05, **p < 0.01, ***p < 0.001

#### Testing the moderating effect of spiritual leadership

Hierarchical regression analysis and mean-centered variables were used to test the moderating effect of spiritual leadership’s various aspects (vision, hope/faith, and altruistic love) on the relationship between compassion fatigue and turnover intention. The results are presented in Tables 5–7. The estimations of all Models 2 in Tables 5–7 revealed that compassion fatigue still significantly and positively influenced turnover intention after being centralized, offering additional support for H3. Furthermore, as Models 3 in Tables 5–7 indicated, compassion fatigue and each dimension of spiritual leadership (vision, hope/faith, and altruistic love) significantly influenced nurses’ turnover intention. However, from the same Models, it can be observed that the interactions of compassion fatigue and vision (β = -0.169, p < 0.001) and altruistic love (β = 0.193, p < 0.01) were statistically significant. Still, the interaction between compassion fatigue and the dimension hope/faith (β = -0.021, p > 0.05) was insignificant. These findings suggest that the subscales vision and altruistic love could moderate the relationship between compassion fatigue and turnover intention but not hope/faith. Hence, offering support for H5a and H5c but not for H5b.

**Table 5:** Hierarchical regression results of the moderating effect of vision on compassion fatigue and turnover intention relationship.

Variables	Turnover intention	Turnover intention	Turnover intention
	Model 1 *β* (t)	Model 2 *β* (t)	Model 3 *β* (t)
(Constant)	4.266*** (6.196)	4.426*** (6.92)	4.427*** (6.915)
Gender	0.064 (0.294)	0.026 (0.128)	0.027 (0.132)
Age	-0.065 (-0.699)	-0.04 (-0.471)	-0.042 (-0.483)
Educational Level	-0.05 (-0.344)	-0.037 (-0.274)	-0.035 (-0.26)
Work Experience	-0.072 (-0.63)	-0.127 (-1.192)	-0.127 (-1.184)
Compassion fatigue		0.380*** (5.494)	0.326*** (5.110)
Vision		-0.218*** (-5.646)	-0.169*** (-1.643)
Compassion fatigue x Vision			-0.087* (-2.231)
R^2^	0.003	0.144	0.153
Δ R^2^	0.003	0.141	0.009
F	0.435	24.569***	18.481***

**Notes**: *β*, Unstandardized estimates; (t), t-values; *p < 0.05, **p < 0.01, ***p < 0.001

**Table 6:** Hierarchical regression results of the moderating effect of hope/faith on compassion fatigue and turnover intention relationship.

Variables	Turnover intention	Turnover intention	Turnover intention
	Model 1 *β* (t)	Model 2 *β* (t)	Model 3 *β* (t)
(Constant)	4.266*** (6.196)	4.313*** (6.812)	4.316*** (6.812)
Gender	0.064 (0.294)	0.091 (0.455)	0.09 (0.452)
Age	-0.065 (-0.699)	-0.029 (-0.335)	-0.029 (-0.337)
Educational Level	-0.05 (-0.344)	-0.008 (-0.062)	-0.013 (-0.094)
Work Experience	-0.072 (-0.63)	-0.139 (-1.316)	-0.141 (-1.329)
Compassion fatigue		0.329*** (4.685)	0.309*** (4.561)
Hope/Faith		-0.281*** (-6.542)	-0.246*** (-6.32)
Compassion fatigue x Hope/Faith			-0.021 (-1.021)
R^2^	0.003	0.161	0.166
Δ R^2^	0.003	0.157	0.005
F	0.435	16.572***	14.224***

**Notes**: *β*, Unstandardized estimates; (t), t-values; *p < 0.05, **p < 0.01, ***p < 0.001

**Table 7:** Hierarchical regression results of the moderating effect of altruistic love on compassion fatigue and turnover intention relationship.

Variables	Turnover intention	Turnover intention	Turnover intention
	Model 1 *β* (t)	Model 2 *β* (t)	Model 3 *β* (t)
(Constant)	4.266*** (6.196)	4.429*** (6.882)	4.431*** (6.899)
Gender	0.064 (0.294)	0.032 (0.156)	0.038 (0.188)
Age	-0.065 (-0.699)	-0.052 (-0.599)	-0.052 (-0.603)
Educational Level	-0.05 (-0.344)	-0.014 (-0.102)	-0.012 (-0.089)
Work Experience	-0.072 (-0.63)	-0.136 (-1.264)	-0.132 (-1.231)
Compassion fatigue		0.407*** (5.9)	0.387*** (5.548)
Altruistic love		-0.218*** (-5.011)	-0.22*** (-5.05)
Compassion fatigue x Altruistic love			0.139** (3.658)
R^2^	0.003	0.133	0.149
Δ R^2^	0.003	0.13	0.016
F	0.435	31.93***	23.327 ***

**Notes**: *β*, Unstandardized estimates; (t), t-values; *p < 0.05, **p < 0.01, ***p < 0.001

## Discussion and implications

The Covid-19 pandemic has created realities that considerably influence nurses’ working conditions. These new challenges require adjustments in the organizational life of medical companies [55]. Along this same line, this study relied on the Job Demands-Resources theory to explore the effect of role demands on turnover intention and then through compassion fatigue. As a common practice [8, 60], when dimensions are highly correlated with each other, they can be combined. Therefore, the different aspects of role demands (role conflict and role ambiguity) were combined under one factor, as well as those of compassion fatigue (burnout and secondary trauma), to get the model that best fits the data set. We also investigated how spiritual leadership, with its various dimensions (vision, hope/faith, altruistic love), could moderate the relationship between compassion fatigue and turnover intention.

### Theoretical implications

The findings of this research contribute to the extant literature by elaborating on the likelihood that conflicting and ambiguous work roles with limited resources can affect employees’ well-being and cause them to consider leaving their organization. This research also answered the call of previous studies by Reference [7] and Reference [1] to extend similar approaches in other provinces in China to understand better the causing variables of turnover intention among nurses. Finally, our findings bring additional support to the assumptions of the JD-R theory.

The positive and significant impact of role demands on turnover intention was similarly proven in previous research, demonstrating that inadequate working conditions, such as stressful roles, can negatively impact workers’ intention to leave [7, 38, 66]. Indeed, in the context of excessive job demands, conflicting and unclear roles may frustrate and impede employees from executing their job tasks successfully, fueling their desire to consider alternative employment [67].

Role demands could significantly impact compassion fatigue, corroborating Wells’s finding [36]. Working in a severe and risky environment such as Covid-19 is already challenging for nurses. If added to that, their roles involve unpredictability and incompatibility, then the impact on their health and productivity can only be devastating. Compassion fatigue manifests through burnout and traumatic stress from working with suffering patients. Burnout and mental health problems have been linked to role conflict and ambiguity [38, 68]. It is inappropriate to work in an environment where it is possible to face competitive or contradictory demands and the job scope, parameters, or goals are not well defined [68].

Compassion fatigue was also found to influence turnover intention significantly and to mediate its relationship with role demands. It has been discussed that compassion fatigue will enhance depersonalization, anxiety, and exhaustion in workers, negatively influencing their performance and organizational behaviors [42]. In addition, long-term experience of compassion fatigue has been demonstrated to affect employees’ caregiving abilities, threatening their satisfaction and engagement, generally buffering their negative feelings to turn into turnover intentions [41]. Moreover, compassion fatigue encapsulating burnout has been reported to mediate the relationship between role demands and turnover intention in this study. This outcome aligns with the JD-R theory that proposed burnout as a mediator between job demands, job resources, and turnover intention [23]. The precedent suggests that a negative psychological state, such as burnout or secondary trauma, created by conflicting or ambiguous work roles and inadequate resources might instigate turnover intentions in workers.

Spiritual leadership was shown to moderate the relationship between compassion fatigue and turnover intention through vision and altruistic love but not through hope/faith. These results were consistent with previous studies suggesting that spiritual leadership affects retention and mental health [17]. As stipulated in the JD-R theory, job resources are inherent motivational qualities that foster individuals’ willingness to put in the additional effort necessary to reduce job demands and attain work goals. As leadership is essential for workers’ performance and well-being [69], applying appropriate leadership could alleviate the adverse influence of burnout and other mental health issues by rebuilding a positive and healthier work environment [17]. Reference [69] argued that leadership could act as a moderator and modify the effect of job demands, job resources, or personal resources on attitudinal outcomes, especially when working conditions are difficult to alter.

For instance, spiritual leadership is supposed to provide leaders and followers with a compelling vision (institution’s fundamental aspiration), where they have that sense of calling (centering on selfless service to clients) to work differently and make their job meaningful. Moreover, it establishes an organizational culture where altruistic love is prone; leaders and followers have that sense of membership, are concerned, and care for each other, making them feel loved, understood, and appreciated [53].

**Fig 2:**
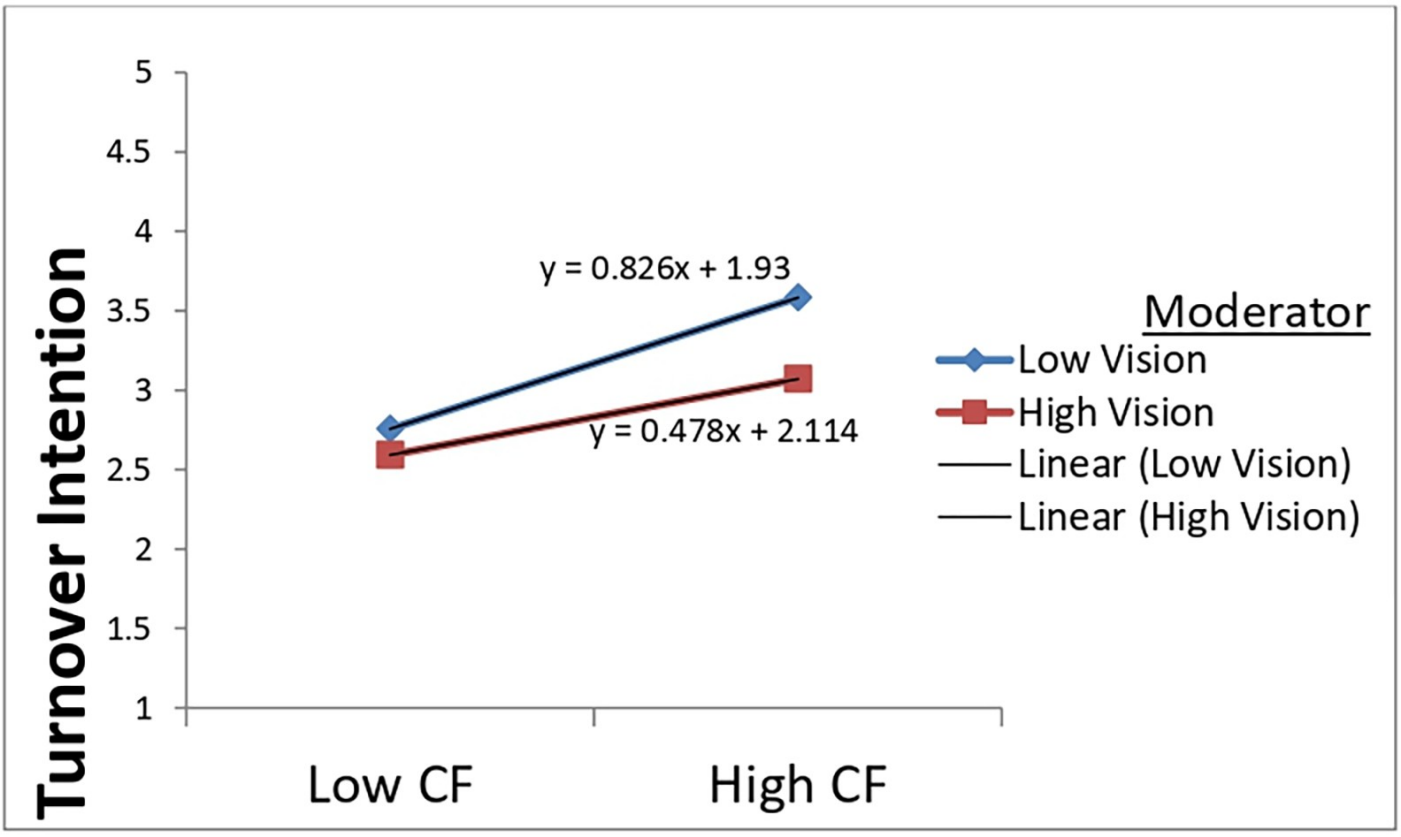
Moderating effect of vision on compassion fatigue and turnover intention relationship.

First, the likelihood that the desire to quit arises from compassion fatigue is lower when employees acknowledge carrying the organization’s vision (See Fig 2).

**Fig 3:**
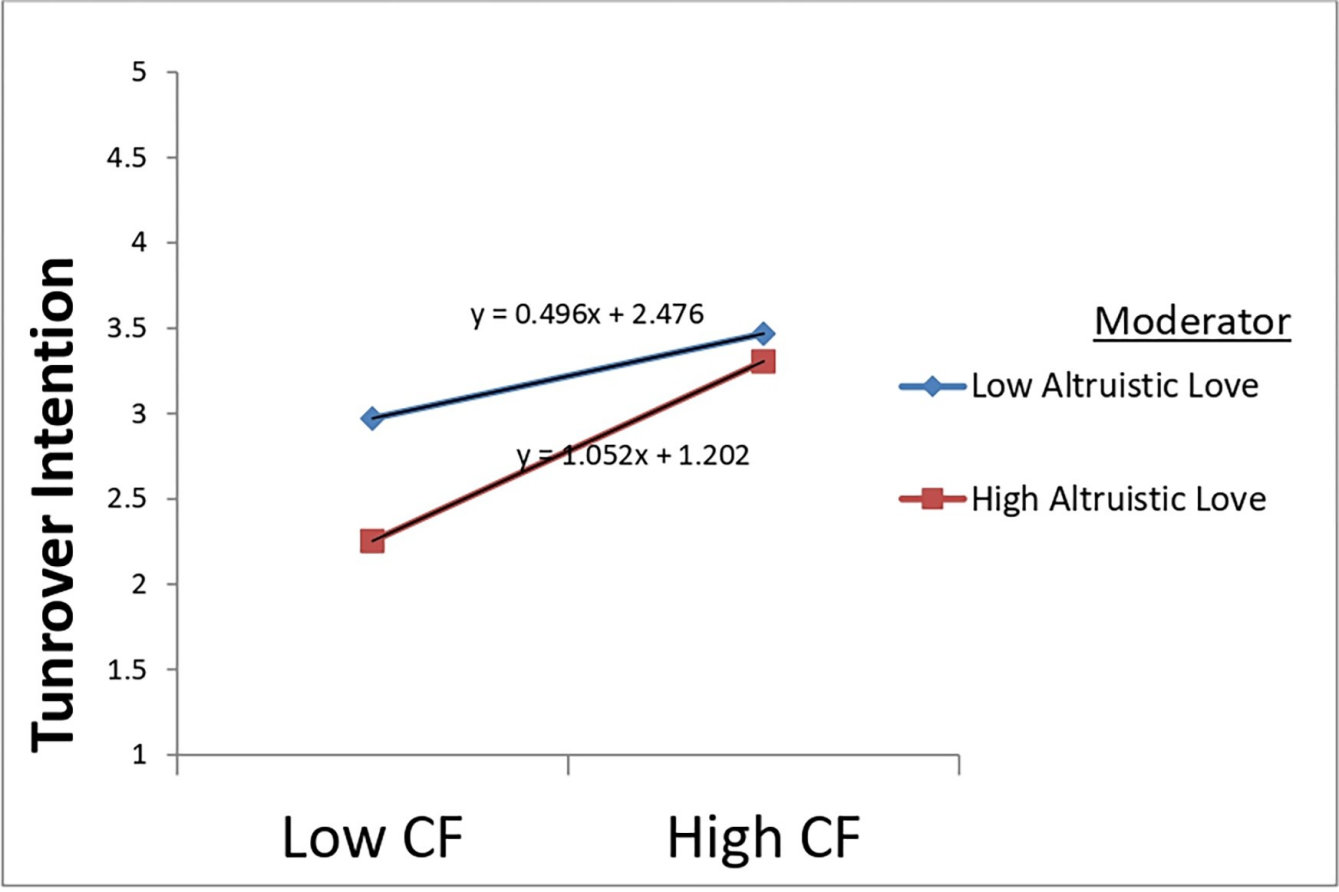
Moderating effect of altruistic love on compassion fatigue and turnover intention relationship.

**Fig 4:**
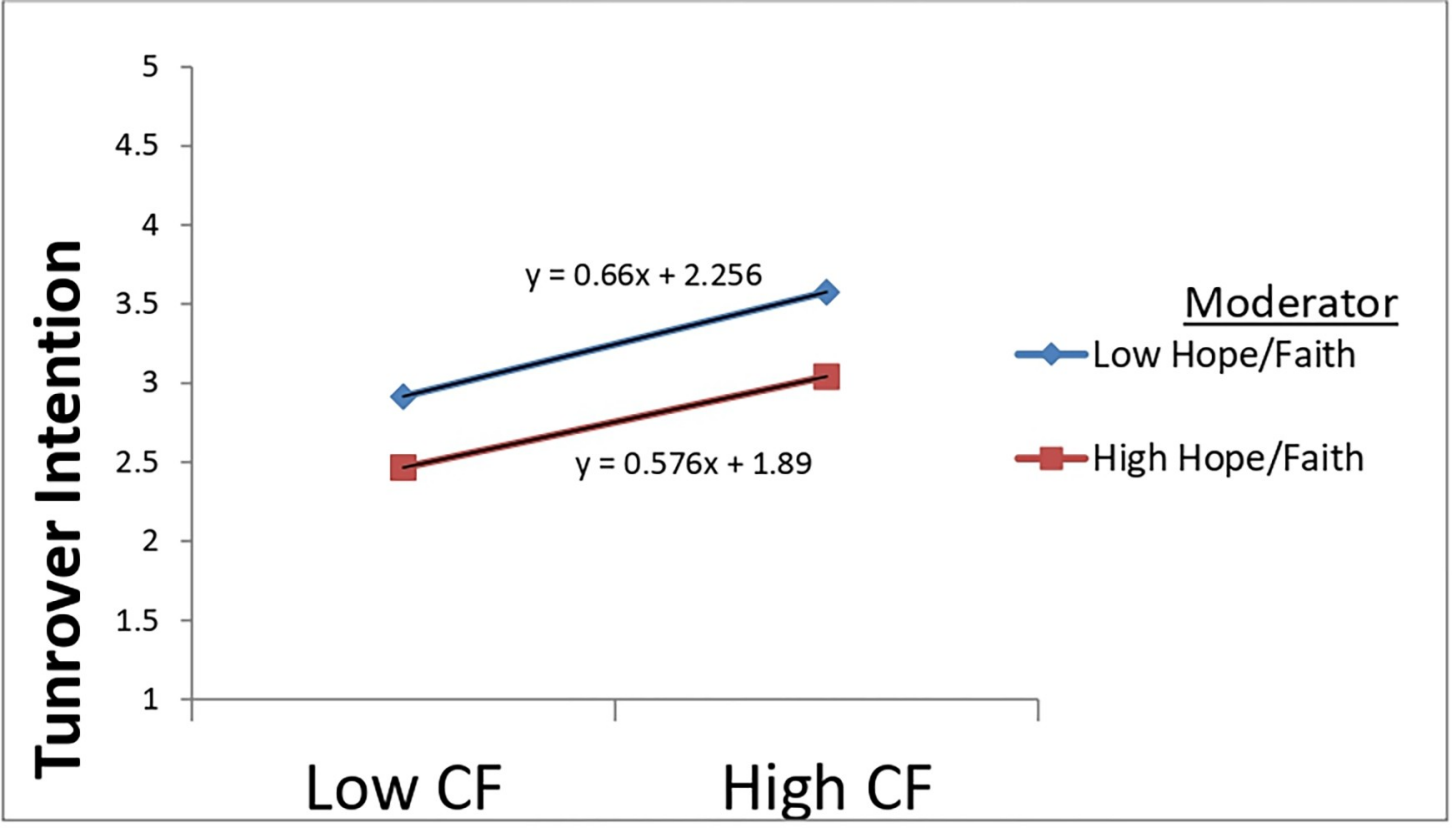
Moderating effect of hope/faith on compassion fatigue and turnover intention relationship.

Furthermore, the continual support from leaders equips them to go beyond themselves and find new ways to perform according to the standards. In that way, they are less likely to plan to leave the company [19]. Second, despite the challenges, an organizational culture characterized by true love, appreciation, and care buffers the negative feelings about the working conditions and diminishes workers’ leaving intent (See Fig 3). Such a working milieu inspires confidence and trust, where employees can share insights into better handling exhaustion and traumatic stress [17, 49]. Third, the fact that the dimension hope/faith could not moderate compassion fatigue and turnover intention relationship was surprising. As shown in Fig 4, at a low level of hope/faith, the relationship between compassion fatigue and turnover intention is stronger. This outcome suggests that Chinese frontline nurses might have lost hope that the situation could get better soon since the coronavirus is still around, making victims, increasing their workload, and consequently threatening their well-being [2, 47].

### Practical implications

This study that examined the interplay of role demands with compassion fatigue and spiritual leadership to predict turnover intention among Chinese frontline nurses has great practical significance. Confusion and uncertainty about one’s job might be a source of stress for nurses. For some employees, admitting their uncertainty might be seen as incompetency, or they could be afraid to receive more responsibilities [50, 67]. Therefore, identifying the aspects of frontline nurses’ confusing job tasks and dealing with them is essential. For example, medical institutions could formalize what is expected from nurses in written policies, implement a feedback mechanism where they feel free to ask for clarification about their job tasks anytime and be transparent about the availability of resources concerning their job’s requirements [67].

The cost of caring puts frontline nurses in a perpetually vulnerable state, and uncertainty in their job does not help that fact [31]. In addition, the emotional strain and the burden of the victims of the Covid-19 pandemic have caused more exhaustion and dysfunction among frontline nurses [29, 39]. Thus, training frontline nurses in recognizing the causes, risk factors, and development of compassion fatigue is also critical. Knowing more about compassion fatigue will facilitate establishing a peer support system that enables nurses to prevent and deal with it. Hospital leaders could also put in place assistance programs to alleviate frontline nurses’ emotional burdens due to their work.

Good leadership can influence job demands and resources directly or as a moderator. Their impact on job characteristics could affect job crafting and self-undermining [69]. Along this line, implementing spiritual leadership will work as additional energy or fuel to enable nurses to align their perception with their organization’s values and be more passionate and fulfilled at work [19]. In addition, emphasizing nurses’ spiritual well-being might benefit healthcare institutions because their sense of calling will magnify their purpose and personal fulfillment. In addition, their sense of membership will foster team collaboration, which is essential for the effective delivery of care [17]. Therefore, healthcare leaders must encourage nurses’ inner life and mindful practices that might be a source of strength to fortify them in times of need. They can also cultivate an organizational culture based on genuine love and support to see each team member thrive in their work, even in difficult times, and most importantly, stay in their jobs. [53].

### Limitations

The present study has some limitations that can lead to further research opportunities. Data were collected in second and third-level hospitals. Although the patient care protocol is more or less harmonized and homogeneous in the selected hospitals, future research may consider including other contextual and individual factors related to the variables involved in the contextual model. Despite the importance of our findings, the descriptive nature of the study makes it difficult to claim causality. A longitudinal design may better provide a causal interpretation of the relationship between the variables.

Moreover, alternative factors might be used to mediate role demands and turnover intention or moderate the positive effect of compassion fatigue on turnover intention. For example, the revised JD-R model considered engagement an antipode of burnout and suggested it mediates job demands and health problems as well as job resources and turnover intention [23, 70]. Other buffering factors might also help to identify internal stakeholders who can help nurses to perform adequately even in the presence of unclear guidelines and a weak mental state.

## Conclusion

Since the beginning of the Covid-19 pandemic, healthcare practitioners have worked hard to offer patients the necessary care delivery. However, the saturation of medical institutions exposed the need to increase labor costs. The current discourse on turnover intention and its substantial influencing variables, such as role demands, compassion fatigue, and spiritual leadership, has allowed us to dive into the hard time frontline nurses have been going through in recent years. The results highlighted that ambiguous and conflicting roles in a challenging working environment exacerbate frontline nurses’ well-being and expose them to exhaustion and trauma, making them vulnerable and full of negative feelings about their job. The findings also revealed that appropriate leadership, such as spiritual leadership, could dampen the adverse effect of compassion fatigue on turnover intention by providing nurses with additional resources. These research outcomes can serve as a steppingstone for future investigations of how medical institutions can improve nurses’ subjective well-being and increase retention.

## Supporting information

S1 Checklist STROBE statement—checklist of items that should be included in reports of observational studies. (DOCX)

S1 Appendix Questionnaire for the study. (DOCX)
