# Supplementary material for: Role demands and turnover intention among Covid-19 frontline nurses: The mediating and moderating roles of compassion fatigue and spiritual leadership
Source: PLoS One. 2023 Aug 10;18(8):e0289888. doi: 10.1371/journal.pone.0289888 (PMC10414576; doi:10.1371/journal.pone.0289888)
Supplement: S1 Appendix — (DOCX) [file pone.0289888.s002.docx]

**S1 Appendix.** **Questionnaire for the study.**

| **Constructs** | **Description** |
| --- | --- |
| Role Demands (RD) |  |
| 1.Role Ambiguity (RA) |  |
| RA1 | I have clear, planned goals and objectives for my job |
| RA2 | I know that I have divided my time properly |
| RA3 | I know what my responsibilities are |
| RA4 | I know exactly what is expected of me |
| RA5 | I feel certain about how much authority I have on the job |
| RA6 | Explanation is clear of what has to be done |
| 2. Role Conflict (RC) |  |
| RC1 | I have to do things that should be done differently under different conditions |
| RC2 | I receive an assignment without the manpower to complete it |
| RC3 | I have to buck a rule or policy in order to carry out an assignment |
| RC4 | I work with two or more groups who operate quite differently |
| RC5 | I receive incompatible requests from two or more people |
| RC6 | I do things that are apt to be accepted by one person and not by others |
| RC7 | I receive an assignment without adequate resources and materials to execute it |
| RC8 | I work on unnecessary things |
| Compassion Fatigue (CF) |  |
| 1.Secondary Trauma (ST) |  |
| ST1 | Flashbacks connected to clients |
| ST2 | Troubling dreams similar to client’s |
| ST3 | Intrusive thoughts after working with difficult clients |
| ST4 | Suddenly recalled frightening experience while working with client |
| ST5 | Losing sleep over client’s traumatic experience |
| ST1 | Flashbacks connected to clients |
| ST2 | Troubling dreams similar to client’s |
| 2.Job Burnout (JB) |  |
| JB1 | I have felt trapped by my work |
| JB2 | Sense of hopelessness working with clients |
| JB3 | Felt tired due to work as caregiver |
| JB4 | Felt depressed as a result of work |
| JB5 | Unsuccessful at separating work from personal life |
| JB6 | Sense of worthlessness associated with work |
| JB7 | Feel like a “failure” in work |
| JB8 | Thoughts about not achieving goals |

**S1 Appendix** **Questionnaire for the study (Continued).**

| **Constructs** | **Description** |
| --- | --- |
| Spiritual Leadership (SL) |  |
| 1.Vision (VI) |  |
| VI1 | I understand and am committed to my organization’s vision |
| VI2 | My work-group has a vision statement that brings out the best in me |
| VI3 | My organization’s vision inspires my best performance |
| VI4 | I have faith in my organization’s vision for its employees |
| VI5 | My organization’s vision is clear and compelling to me |
| 2.Hope/Faith (HF) |  |
| HF1 | I have faith in my organization and I am willing to “do whatever it takes” to ensure that it accomplishes its mission |
| HF2 | I persevere and exert extra effort to help my organization succeed because I have faith in what it stands for |
| HF3 | I always do my best in my work because I have faith in my organization and its leaders |
| HF4 | I set challenging goals for my work because I have faith in my organization and want us to succeed |
| HF5 | I demonstrate my faith in my organization and its mission by doing everything I can to help us succeed |
| 3.Altruistic Love (AL) |  |
| AL1 | My organization really cares about its people |
| AL2 | My organization is kind and considerate toward its workers, and when they are suffering, wants to do something about it |
| AL3 | The leaders in my organization “walk the walk” as well as “talk the talk” |
| AL4 | My organization is trustworthy and loyal to its employees |
| AL5 | My organization does not punish honest mistakes |
| AL6 | The leaders in my organization are honest and without false pride |
| AL7 | The leaders in my organization have the courage to stand up for their people |
| Turnover Intention (TI) |  |
| TI1 | How likely is that you will actively look for a new job in the next year? |
| TI2 | I often think about leaving the organization |
| TI3 | I will probably look for a new job in the next year |
